# Hospital-Acquired Hyponatremia in Children Following Hypotonic versus Isotonic Intravenous Fluids Infusion

**DOI:** 10.3390/children5100139

**Published:** 2018-10-02

**Authors:** Spyridon A. Karageorgos, Panagiotis Kratimenos, Ashley Landicho, Joshua Haratz, Louis Argentine, Amit Jain, Andrew D. McInnes, Margaret Fisher, Ioannis Koutroulis

**Affiliations:** 1 Division of Infectious Diseases and Center for Childhood Cancer Research, Children’s Hospital of Philadelphia, Perelman School of Medicine at the University of Pennsylvania, Philadelphia, PA 19104, USA; spiroskarageorgo@gmail.com; 2 Division of Neonatology, Children’s National Medical Center, George Washington University School of Medicine and Health Sciences, Washington, DC 20010, USA; Panagiotis.Kratimenos@childrensnational.org; 3 Crozer-Chester Medical Center, Crozer-Keystone Health Network, Upland, PA 19013, USA; Ashley.landicho@gmail.com; 4 St. Christopher’s Hospital for Children, Drexel University College of Medicine, Philadelphia, PA 19134, USA; Joshua.haratz@gmail.com (J.H.); ljargentine27@gmail.com (L.A.); 5 Sanford Children’s Hospital, Sanford School of Medicine, University of South Dakota, Sioux Falls, SD 57105; USA; amit.jainms@gmail.com; 6 K. Hovnanian Children’s Hospital, Jersey Shore University Medical Center, Neptune, NJ 07753, USA; Andrew.mcinnes@hackensackmeridian.org; 7 The Unterberg Children’s Hospital, Monmouth Medical Center, Drexel University College of Medicine, Long Branch, NJ 07740, USA; Margaret.Fisher@rwjbh.org; 8 Division of Emergency Medicine, Children’s National Medical Center, George Washington University School of Medicine and Health Sciences, Washington, DC 20010, USA

**Keywords:** hospital-acquired, hyponatremia, pediatrics, parenteral solutions, hypotonic fluids, isotonic fluids

## Abstract

Hypotonic solutions have been used in pediatrics for maintenance of intravenous (IV) hydration. However, recent randomized control trials and cohort studies have raised significant concerns for association with hospital-acquired hyponatremia (HAH). The study aimed to assess whether the use of hypotonic parenteral solutions (PS) compared with isotonic PS is associated with increased HAH risk in children with common pediatric conditions. Retrospective chart review of 472 patients aged 2 months to 18 years who received either isotonic or hypotonic PS as maintenance fluids. Administration of hypotonic PS was associated with a four-fold increase in risk of developing HAH in the univariate analysis, (unadjusted odds ratio (OR) = 3.99; 95% confidence interval (CI): 1.36–11.69, *p =* 0.01). Hypotonic PS were associated with HAH (*p* = 0.04) when adjusted for the level of admission serum CO_2_. There was a mean decrease of serum sodium of 0.53 mEq/L in the hypotonic group compared to the mean increase of 4.88 mEq/L in the isotonic group. These data suggest that hypotonic PS are associated with HAH in children admitted for common pediatric conditions. Isotonic PS should be considered as a safer choice for maintenance fluid hydration.

## 1. Introduction

Hospital-acquired hyponatremia (HAH) is an electrolyte imbalance affecting as many as 25% to 40% of hospitalized pediatric patients [1]. Severe hyponatremia can result in cerebral edema and neurologic dysfunction including brain damage, seizures, respiratory arrest, and death [2]. The occurrence of the electrolytic imbalance is often difficult to predict due to the absence of specific early signs or symptoms, especially in young children [3]. Hyponatremia is common among hospitalized pediatric patients due to the stimulation of antidiuretic hormone (ADH) secretion in children under stress, trauma, pain, or nausea/vomiting that many hospitalized children experience [2,4]. Volume depletion (vomiting) constitutes a major stimulus for ADH secretion and can result in hyponatremia [2,4].

Hypotonic fluids became the recommended maintenance fluids for pediatric patients after the Holliday and Segar study in 1957 [5]. In the last decade, several authors have argued that the use of hypotonic versus isotonic maintenance fluids in hospitalized pediatric patients has resulted in high incidence of HAH [6,7,8]. Moreover, a recent randomized controlled trial (RCT) by McNab et al. revealed that isotonic fluids had a significantly decreased risk for hyponatremia compared with hypotonic [9].

The present study tests the hypothesis that hypotonic parenteral solutions (PS) are associated with increased risk of hospital-acquired hyponatremia in children with common pediatric conditions.

## 2. Patients and Methods

### 2.1. Setting and Study Design

A retrospective cohort study was conducted in the Pediatric Emergency and Inpatient Units of St. Christopher’s Hospital for Children, Philadelphia, PA and The Unterberg Children’s Hospital at Monmouth Medical Center, Long Branch, NJ, from 1 January 2010 to 31 December 2012. This study was approved by the Drexel University Institutional Review Board (no. 1307002193) and by the Monmouth Medical Center Institutional Review Board (no. 12-030) and is reported in accordance with STROBE recommendations for cohort studies (supplemental file 1). Both institutions are tertiary pediatric hospitals with the full spectrum of pediatric subspecialties [10].

The study cohort included all patients between 2 months to 18 years of age who received either isotonic or hypotonic parenteral solutions for at least 8 h; and had at a minimum of two basic metabolic panel measurements throughout their admission with a 12 h interval or longer in-between them. Infants younger than 2 months of age; patients with initial serum glucose <50 mg/dL; children with preexisting renal or cardiac failure; children in shock; and children with preexisting metabolic or electrolyte disorders were excluded. For children with multiple admissions that met the inclusion criteria, only the first admission data were extracted and analyzed. Serum sodium was measured with a direct potentiometry method [11].

### 2.2. Definitions and Data Collection

Patients were separated into three groups according to serum sodium levels: Na ≤ 135 mEq/L (hyponatremia), Na = 136–144 mEq/L (normal sodium level), and Na ≥ 145 mEq/L (hypernatremia). Maintenance intravenous (IV) parenteral solutions were categorized under three classifications: 1 = isotonic parenteral solution, 2 = hypotonic parenteral solutions (½ normal saline (NS)), and 3 = other hypotonic parenteral solutions. Isotonic parenteral solutions used for maintenance fluids included 0.9% sodium chloride (NS), 5% dextrose in 0.9% sodium chloride (D5NS), 10% dextrose in 0.9% sodium chloride (D10NS), 12% dextrose in 0.9% sodium chloride (D12NS). Hypotonic parenteral solutions used for maintenance fluids included 5% dextrose in 0.45% sodium chloride (D5 ½ NS). Other hypotonic parenteral solutions included 5% dextrose in 0.675% sodium chloride (D5 ¾ NS), and 5% dextrose in 0.225% sodium chloride (D5 ¼ NS). Lactated Ringer’s was not used as a solution in any of the patients based on institutional practices. Although 5% dextrose, 10% dextrose, and 12% dextrose added to 0.9% sodium chloride or 0.45% sodium chloride are chemically classified as hypertonic and hypotonic solutions, respectively, the dextrose portion of the solution is metabolized quickly; therefore, only the osmolarity of the underlying solution was considered. The initial and last serum carbon dioxide (CO_2_) levels were used for analysis, with no serum quantity categorization.

All data were extracted from St. Christopher’s Hospital for Children and the Children’s Hospital at Monmouth Medical Center EMR (HPF, Tenet Healthcare Corporation, Dallas, TX, USA). Data collected from chart analysis were stored on an online database, Research Electronic Data Capture (REDCap), using non-identifiable records and password protection [12].

### 2.3. Statistical Analysis

Predictors of HAH were evaluated using univariate and multiple variable logistic regression with significant predictors from the univariate analysis (*p* < 0.10) entered into the multiple variable model using backward elimination. The final model included significant variables (*p* < 0.05) adjusted for the other variables. Odds ratios (OR) with 95% confidence intervals (CI) were also included. All data analysis was carried out using Statistical Analysis System (SAS) v9.4 software (SAS Institute, Cary, NC, USA).

## 3. Results

### 3.1. Baseline Characteristics of Included Patients

The final study cohort contained 472 patients (227 males, 245 females) that met the inclusion criteria. Initial diagnoses were vomiting/gastroenteritis (*n* = 367), pyloric stenosis (*n* = 76), pneumonia (*n* = 17), bronchiolitis/asthma (*n* = 9), and appendicitis (*n* = 3). All patients presented with dehydration which necessitated the use of IV fluids and were admitted to the Pediatric Inpatient Unit for further management. Intravenus fluids were administered as per institution protocols.

### 3.2. Clinical Characteristics and Outcomes

Of the 472 patients, 63% (*n* = 299) received isotonic maintenance fluids and 37% (*n* = 173) received hypotonic maintenance fluids. A total of 273 patients (128 in the isotonic and 145 in the hypotonic group) had a normal admission serum sodium value within 136–144 mEq/L, 5.49% of which developed hyponatremia in their hospital stay. Also, 180 patients had hyponatremia on admission with Na ≤ 135 mEq/L, without clinical manifestations of hyponatremia, and 18.89% of which had persistent hyponatremia during their hospital stay. Results from the multiple variable logistic regression model are shown in Table 1.

Administration of hypotonic PS was associated with a four-fold increase in risk of developing HAH in the univariate analysis (unadjusted OR = 3.99; 95% CI: 1.36–11.69, *p =* 0.01). Administration of hypotonic PS remained independently associated with the development of HAH in the multiple variable analysis (adjusted OR = 3.14; 95% CI: 1.03–9.53, *p =* 0.4) when adjusted for admission CO_2_. Each unit decrease in admission CO_2_ was associated with a 5.0% increase in the risk of HAH (adjusted OR = 1.05; 95% CI: 1.01–1.09, *p* < 0.1).

**Figure 1:**
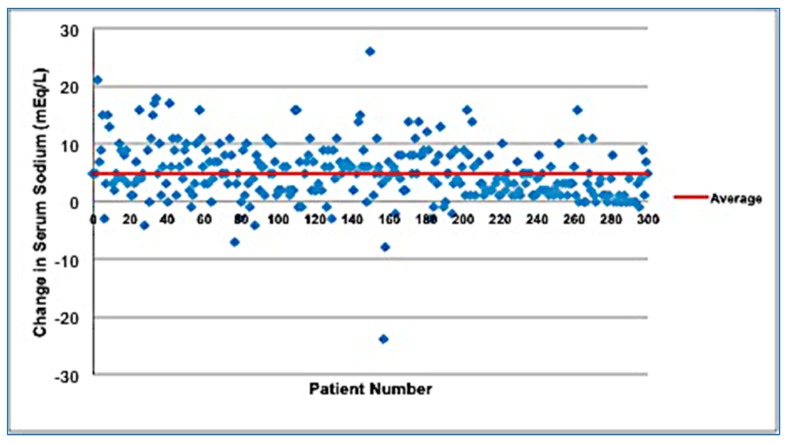
Change in serum sodium levels (mEq/L) in patients that received isotonic maintenance intravenous (IV) fluids. Note that there was an increase in the mean serum sodium concentration by approximately 4.88 mEq/L following the administration of isotonic parenteral solutions (PS).

**Figure 2:**
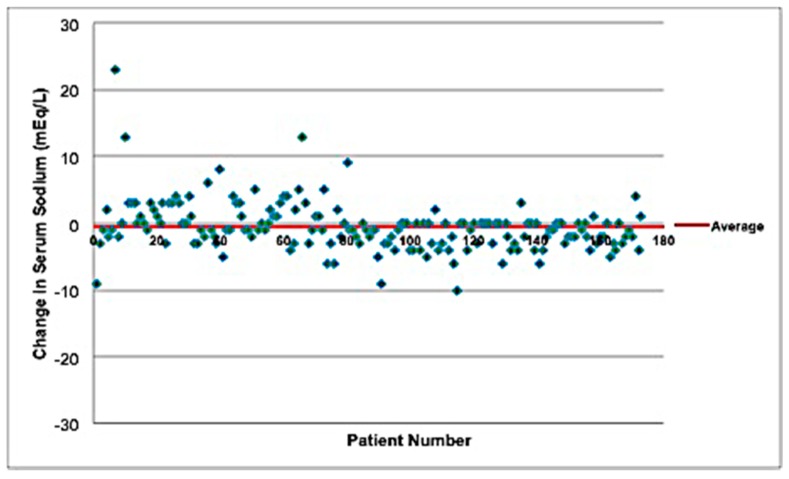
Change in serum sodium levels (mEq/L) in patients that received hypotonic maintenance IV fluids. Changes in serum sodium concentration (mEq/L) following the administration of hypotonic PS. Note that there was a decrease in the mean serum sodium concentration by 0.53 mEq/L following the administration of hypotonic PS.

Changes in serum sodium levels after isotonic and hypotonic maintenance fluids are presented in Figure 1 and Figure 2, respectively. Subjects who received isotonic PS had a mean increase of serum sodium of 4.88 mEq/L, while those who received hypotonic PS had a mean decrease of serum sodium of 0.53 mEq/L. For all participants, the admission sodium serum range was 116–155 mEq/L, while the discharge sodium range was 129–149 mEq/L. For those who received isotonic PS, the range of serum sodium change was from −24 to 26 mEq/L, while those who received hypotonic PS, the serum sodium change was −10 to 23 mEq/L. For those who received isotonic PS, the range of admission CO_2_ was 2 to 49 mEq/L, while those who received hypotonic PS, the range of admission CO_2_ was 5 to 48 mEq/L. Both groups were similar in terms of the amount of administered bolus hydration (84% in the isotonic PS versus 86% in the hypotonic PS group).

## 4. Discussion

Since the study by Holliday and Segar in 1957, hypotonic solutions have been used for pediatric patients requiring maintenance IV hydration [5]. However, there is a growing body of evidence supporting the association of hypotonic PS with electrolytic abnormalities, such as HAH, which may carry significant morbidity and mortality [3,13].

In the present study, we showed that pediatric patients receiving hypotonic PS had an increased risk of developing HAH compared to patients receiving isotonic PS, without a statistically significant change in their other serum electrolyte levels. The results are in line with other observational studies and smaller randomized controlled trials which highlighted a relationship between HAH and hypotonic solutions [14,15,16]. Also, a recent RCT which included 690 children revealed that isotonic intravenous fluids had a statistically significant decreased risk for hyponatremia compared to hypotonic fluids without any difference regarding observed adverse events [17].

Moreover, our findings are consistent with the existent literature that describes HAH in hospitalized pediatric patients within a wide range of clinical settings with varying rates of fluid administration, surgical versus medical patients, or isolated groups such as critically-ill patients [6,18]. The odds ratio of 3.14 from the multivariate analysis is similar to the calculated odds ratio of 3.49 (relative risk (RR) = 2.24) from the meta-analysis by Wang et al. which included ten RCTs that compared isotonic to hypotonic fluid therapy in hospitalized children [18]. Our findings showed a slightly greater, but still consistent, odds ratio with Foster’s meta-analysis of ten RCT studies comparing hypotonic versus isotonic maintenance fluids in hospitalized children with an overall relative risk of hyponatremia of 2.37 (95% CI: 1.72–3.26) [19]. Also, McNab’s meta-analysis showed that the use of isotonic fluids reduced by half the risk of hyponatremia (RR = 0.48), compared with hypotonic fluids [9].

Our data show that every 1 mEq/L decrease in CO_2_ on admission was associated with a 5.0% increase in the risk of HAH. The association between the serum CO_2_ with serum sodium remains unclear, however, there is some evidence supporting a correlation between hyponatremic patients associated with adrenocorticotropic deficiency (ACTH) and low serum HCO_3_ levels [20]. The data indicate that hypotonic PS resulted in a serum sodium change of −0.53 mEq/L, which is less than the serum change of −3.22 mEq/L found by the study in Rey et al. [16]. This disparity may be due to the fact that in the abovementioned study included critically ill children who were already at risk for hyponatremia on admission.

The strengths of the current study lie in its comprehensive coverage of a wide set of common pediatric conditions within the inpatient general pediatric cohort over a longer period duration. We included patients in the general pediatric ward without complex surgical or medical issues that received PS mostly due to moderate hydration. Having a consistent, informatics-enabled extraction of data from two separate medical center databases bolstered these results. The limitations of this study include intergroup differences that were not accounted for in patients in both parenteral solution groups. Unadjusted patient disparities that include age, severity, or type of illness, and duration of hospital stay could affect HAH risk rates. Also, gastroenteritis could be a possible confounding factor for electrolyte changes. In addition, volume and rate of administered fluids were not determined and considered within our statistical analysis, and therefore evaluation of its effect on HAH could not be determined. However, in several studies, the sole significant predictor of hyponatremia in sick and postoperative children was the fluid type, more than the actual rate of infusion [21,22]. It is also important to note that this study was designed to analyze the intermediate outcome of serum sodium changes, and not clinically relevant outcomes or complications. This was done because power would be limited; a study focusing on clinical outcomes would require very large sample sizes because of complication rarity.

## 5. Conclusions

In summary, we show herein that there is an association between the administration of hypotonic PS for maintenance hydration with the development of HAH in hospitalized pediatric patients. Although switching to isotonic maintenance fluids could eliminate the risk for HAH, our data suggest that there are other factors, such as low serum CO_2_, which may contribute to increased risk for HAH.

We propose that in pediatric wards, intravenous fluids should be administered with caution since both isotonic and hypotonic PS may be associated with complications even following a short duration of treatment. Moreover, the individualization of IV fluids administration and close serum electrolytes monitoring is of significant importance in order to avoid iatrogenic electrolyte disturbances. Larger cohorts and RCTs should focus on the safety of parenteral fluids, examine clinically adverse outcomes, and consider additional risk factors.

## Figures and Tables

**Table 1:** Multiple variable logistic regression model results for development of hospital-acquired hyponatremia (HAH).

Effect	*p*-Value	Odds Ratio *	95% Confidence Limits	
Hypotonic PS	0.04	3.14	1.03	9.53
Admission CO_2_	<0.01	1.05	1.01	1.09

* Odds ratio adjusted for statistically significant variables in the multiple variable model. PS: parenteral solutions.

## Supplementary Materials

The following are available online at http://www.mdpi.com/2227-9067/5/10/139/s1. File 1: STROBE checklist for cohort studies.
