# Supplementary material for: Hospital-Acquired Hyponatremia in Children Following Hypotonic versus Isotonic Intravenous Fluids Infusion
Source: Children (Basel). 2018 Oct 2;5(10):139. doi: 10.3390/children5100139 (PMC6209932; doi:10.3390/children5100139)
Supplement: Supplementary file 1 [file children-05-00139-s001.pdf]

STROBE Statement—Checklist of items that should be included in reports of *cohort studies*

Article title: Hospital Acquired Hyponatremia in Children following Hypotonic versus Isotonic Intravenous Fluids Infusion.

	Item No	Recommendation
**Title and abstract**	1	(*a*) Indicate the study's design with a commonly used term in the title or the abstract<br>**Indicated in abstract.**
		(*b*) Provide in the abstract an informative and balanced summary of what was done and what was found<br>**Done.**
**Introduction**		
Background/rationale	2	Explain the scientific background and rationale for the investigation being reported<br>**Done.**
Objectives	3	State specific objectives, including any prespecified hypotheses<br>**Done.**
**Methods**		
Study design	4	Present key elements of study design early in the paper<br>**Methods section, *Setting and study design.***
Setting	5	Describe the setting, locations, and relevant dates, including periods of recruitment, exposure, follow-up, and data collection<br>**Methods section, *Definitions and data collection.***
Participants	6	(*a*) Give the eligibility criteria, and the sources and methods of selection of participants. Describe methods of follow-up<br>**Methods section, *Setting and study design and Definitions and data collection***
		(*b*) For matched studies, give matching criteria and number of exposed and unexposed<br>**Not applicable.**
Variables	7	Clearly define all outcomes, exposures, predictors, potential confounders, and effect modifiers. Give diagnostic criteria, if applicable<br>**Methods section, *Statistical analysis.***
Data sources/ measurement	8*	For each variable of interest, give sources of data and details of methods of assessment (measurement). Describe comparability of assessment methods if there is more than one group<br>**Methods section, *Definitions and data collection.***
Bias	9	Describe any efforts to address potential sources of bias<br>**Methods section, *Statistical analysis.***
Study size	10	Explain how the study size was arrived at<br>**A sample size calculation was not performed. All eligible patients during the study period were included.**
Quantitative variables	11	Explain how quantitative variables were handled in the analyses. If applicable, describe which groupings were chosen and why<br>**Not applicable.**
Statistical methods	12	(*a*) Describe all statistical methods, including those used to control for

		confounding<br>**Methods section,** ***Statistical analysis.***
		(*b*) Describe any methods used to examine subgroups and interactions<br>**Methods section,** ***Statistical analysis.***
		(*c*) Explain how missing data were addressed<br>**Not applicable.**
		(*d*) If applicable, explain how loss to follow-up was addressed<br>**Not applicable.**
		(*e*) Describe any sensitivity analyses<br>**Methods section,** ***Statistical analysis.***
**Results**		
Participants	13 *	(a) Report numbers of individuals at each stage of study—e.g., numbers potentially eligible, examined for eligibility, confirmed eligible, included in the study, completing follow-up, and analysed<br>**The final study cohort contained 472 patients (227 males, 245 females) that met the inclusion criteria. Initial diagnosis were vomiting/gastroenteritis (*n* = 367), pyloric stenosis (*n* = 76), pneumonia (*n* = 17), bronchiolitis/asthma (*n* = 9) and appendicitis (*n* = 3). All patients independently of the initial diagnosis presented with dehydration which necessitated the use of IV fluids. All patients were admitted in Pediatric Inpatient Unit for further management.**
		(b) Give reasons for non-participation at each stage<br>**Not applicable.**
		(c) Consider use of a flow diagram
Descriptive data	14*	(a) Give characteristics of study participants (e.g., demographic, clinical, social) and information on exposures and potential confounders<br>**Results section,** ***Baseline characteristics of included patient, Clinical characteristics and outcomes.***
		(b) Indicate number of participants with missing data for each variable of interest<br>**Not applicable.**
		(c) Summarise follow-up time (e.g., average and total amount)<br>**Not applicable.**
Outcome data	15*	Report numbers of outcome events or summary measures over time<br>**Results section,** ***Clinical characteristics and outcomes.***
Main results	16	(*a*) Give unadjusted estimates and, if applicable, confounder-adjusted estimates and their precision (e.g., 95% confidence interval). Make clear which confounders were adjusted for and why they were included<br>**Done.**
		(*b*) Report category boundaries when continuous variables were categorized<br>**Not applicable.**
		(*c*) If relevant, consider translating estimates of relative risk into absolute risk for a meaningful time period<br>**Not applicable.**

Other analyses	17	Report other analyses done—e.g., analyses of subgroups and interactions, and sensitivity analyses<br>**Methods section, *Statistical analysis*.**
**Discussion**		
Key results	18	Summarise key results with reference to study objectives<br>**Discussion section, second paragraph.**
Limitations	19	Discuss limitations of the study, taking into account sources of potential bias or imprecision. Discuss both direction and magnitude of any potential bias<br>**Discussion section, sixth paragraph.**
Interpretation	20	Give a cautious overall interpretation of results considering objectives, limitations, multiplicity of analyses, results from similar studies, and other relevant evidence<br>**Discussion section, sixth paragraph and conclusion.**
Generalisability	21	Discuss the generalisability (external validity) of the study results<br>**Discussion section, sixth paragraph and conclusion.**
**Other information**		
Funding	22	Give the source of funding and the role of the funders for the present study and, if applicable, for the original study on which the present article is based<br>**No funding was used for the study.**

* Give information separately for exposed and unexposed groups.

Note: An Explanation and Elaboration article discusses each checklist item and gives methodological background and published examples of transparent reporting. The STROBE checklist is best used in conjunction with this article (freely available on the Web sites of PLoS Medicine at http://www.plosmedicine.org/, Annals of Internal Medicine at http://www.annals.org/, and Epidemiology at http://www.epidem.com/). Information on the STROBE Initiative is available at http://www.strobe-statement.org.
